# Exploring protein glycosylation across GlyGen, UniProtKB, and related knowledgebases for data integration

**DOI:** 10.1093/database/baag064

**Published:** 2026-10-23

**Authors:** Jeet Vora, Kate Warner, Preethi Vasudev, Sandra Orchard, Sujeet Kulkarni, Raja Mazumder, Maria Martin

**Affiliations:** Department of Biochemistry and Molecular Medicine, The George Washington University Medical Center, Washington, DC 20037, United States; Department of Biochemistry and Molecular Medicine, The George Washington University Medical Center, Washington, DC 20037, United States; European Bioinformatics Institute, Wellcome Trust Genome Campus, Hinxton, Cambridge CB10 1SD, United Kingdom; European Bioinformatics Institute, Wellcome Trust Genome Campus, Hinxton, Cambridge CB10 1SD, United Kingdom; Complex Carbohydrate Research Center, University of Georgia, Athens, GA 30602, United States; Department of Biochemistry and Molecular Medicine, The George Washington University Medical Center, Washington, DC 20037, United States; European Bioinformatics Institute, Wellcome Trust Genome Campus, Hinxton, Cambridge CB10 1SD, United Kingdom

## Abstract

Protein glycosylation plays a crucial role in cellular function, protein stability, and disease mechanisms, making the accurate annotation and integration of glycobiology data essential for biological research. However, the inherent complexity of glycosylation and the heterogeneity of glycans present significant challenges for data integration, annotation, and accessibility across biological databases. In this paper, we explore the protein glycosylation information present in GlyGen, a glycoscience knowledgebase, Universal Protein Resource Knowledgebase (UniProtKB), a proteomics knowledgebase, and other resources such as Chemical Entities of Biological Interest (ChEBI) and MatrixDB from International Molecular Exchange (IMEx). We detail the systematic approaches used to annotate glycoproteins, glycosyltransferases, and glycohydrolases across these databases, highlighting the controlled vocabularies and standardized methodologies employed. The integration of data between these resources enables comprehensive access to glycobiology data, from protein sequence and modification sites to detailed glycan structures and biochemical reactions. We also present tools developed through collaboration with UniProt, including the GlyGen Isoform Mapper and enhanced visualization capabilities through ProtVista, which facilitate the exploration of glycosylation in the context of protein structure and function. Through extensive cross-referencing between glycomics and proteomics resources, we establish a robust, interoperable data ecosystem that supports researchers in studying glycan biosynthesis, protein–glycan interactions, and their broader biological implications. Our work demonstrates how database harmonization and collective efforts can drive advancements in glycobiology, providing researchers with high-quality, interconnected data resources to explore and facilitate biomedical research in health and disease.

## Introduction

Glycans are complex carbohydrate molecules formed from monosaccharides that are covalently linked through glycosidic bonds in numerous ways to produce a range of extended, branched oligosaccharides and polysaccharides. These attach to proteins or lipids and are mainly found on the exterior surface of cells or on secreted macromolecules, where they perform various functions in cellular and organismal biology. For example, at the cellular level, glycans and their macromolecules, contribute to structural interactions between and within cells, act in nutrient storage and sequestration, and the diverse patterns of cellular surface glycans provide biological information for cell–cell recognition (intrinsic recognition) and for cell–microbe recognition (extrinsic recognition) [1–4]. At the level of the protein, glycans can change the biophysical properties of the attached proteins, often ensuring correct folding and function of the glycoproteins, and mediating processes such as protein stability, trafficking, and cell attachment [5, 6]. As a consequence, dysregulation of glycosylation and abnormal glycan structures can result in protein dysfunction, and as glycoproteins have important roles in almost all aspects of cell and organismal biology, the study of protein glycosylation has important implications in almost all fields of biological research, including the study of health, disease, nanotechnology, bioenergy, biomaterials, and biotherapeutics [7–13].

Protein glycosylation, the covalent attachment of glycans to specific residues of target proteins, is a universal and essential protein modification carried out by a series of glycosyltransferases and glycan-processing enzymes. Unlike proteins, glycans are not directly encoded by the genome; instead, assembly of the complex multi-subunit, linear, or branched glycan structures onto a polypeptide can take multiple steps and depend on different classes of enzymes, sugar precursors, and organelle structures. Protein glycosylation mainly takes place in the endoplasmic reticulum or Golgi apparatus and is catalyzed primarily by glycosyltransferase enzymes. The majority of glycosyltransferases encoded by the genome are responsible for elongating the attached glycan chain and assembling the monosaccharide moieties into the linear or branched glycan, with the glycosyltransferase acting sequentially so the products of one enzyme become the substrate for the next glycosyltransferase. Other glycosyltransferases are responsible for identifying the proteins that require glycosylation and catalyzing the transfer of either a mono- or oligosaccharide directly to specific residues within the polypeptide [14]. Glycans can be covalently attached to proteins through several major mechanisms: (i) *N*-linked glycosylation, in which glycans are attached to asparagine (Asn) residues; (ii) *O*-linked glycosylation, in which glycans are attached to serine (Ser), threonine (Thr), and less commonly tyrosine (Tyr) residues; (iii) *C*-mannosylation, in which mannose is attached to tryptophan (Trp) residues; and (iv) *S*-linked glycosylation, in which glycans are attached through the sulphur atom of cysteine (Cys). In addition, some proteins undergo glypiation, in which a glycosylphosphatidylinositol (GPI) anchor is attached to the protein. These glycosylation sites are typically found on the extracellular region of transmembrane proteins or on secreted proteins, although simple glycan structures can also be found on nuclear and cytoplasmic proteins. Another class of glycoenzymes called glycosidases, also known as glycohydrolases, degrade glycan structures for uptake and sugar metabolism. They are also important for glycan biosynthesis by removing monosaccharides to form intermediates used as substrates by glycosyltransferases [15]. In addition, the resulting glycan structure can also undergo further modification by other enzyme classes, such as phosphotransferases, phosphoethanolamine transferases, *O*-acetyltransferases, *O*-methyltransferases, sulphotransferases, and pyruvyl transferases. The process of protein glycosylation is further complicated by microheterogeneity, in that at any glycosylation site on a protein sequence synthesized by a specific cell, the glycan structure can vary and can even be absent, and therefore a protein can have many different ‘glycoforms’ [16].

To unravel the complexity of protein glycosylation, therefore, requires an understanding of both proteomics and glycomics, which are complicated topics in themselves. Over the last two decades, rapid technological advances in proteomics and glycomics have generated an unprecedented increase in the volume and complexity of biological data, driving the development of specialized bioinformatics knowledgebases for the organization, integration, visualization, and interpretation of protein–glycan-related information. These resources play a critical role in enabling researchers to connect protein sequence, structure, molecular interactions, glycan composition, biosynthetic pathways, site-specific glycosylation patterns, disease associations, tissue-specific expression, and functional annotations across diverse datasets, analytical methods, and experimental platforms.

In this paper, we highlight how collaboration, data sharing, and integration between the glycomics knowledgebase GlyGen and the proteomics and chemical knowledgebases, such as Universal Protein Resource Knowledgebase (UniProtKB), MatrixDB from the International Molecular Exchange (IMEx) Consortium, and Chemical Entities of Biological Interest (ChEBI), which are all part of the ELIXIR core data resources, leverage the unique expertise of each database to deliver high-quality, curated information on protein glycosylation and glycans. While other resources may include some glycosylation data, this paper focuses exclusively on these select knowledgebases.

### Knowledgebases supporting glycosylation research

#### UniProtKB

The Universal Protein Resource (UniProt) (https://www.uniprot.org/) suite of protein sequence databases is the world’s leading resource for the description of protein sequences and functions [17]. At its heart lies the UniProt Knowledgebase (UniProtKB), a curated resource that comprises a reviewed protein set (UniProtKB/Swiss-Prot), where each protein entry is linked to a summary of the experimentally verified, or computationally predicted, functional information added and evaluated by an expert biocurator, and an unreviewed protein set (UniProtKB/TrEMBL), in which entries are computationally annotated by automated systems. The source of each piece of information added to a record is clearly indicated, enabling users to use this evidence trail to trace the information back to either the original publication in which an experiment was described, an annotation transfer rule, or the algorithm by which the statement was generated. Information captured for each protein entry may include the role(s) the molecule plays in a specific cell type, its 3D structure, subcellular location, interactions with other molecules, and the presence and downstream consequence of any variation in its amino acid sequence.

Central to our understanding of the function of a protein [18], and how they change in response to external stimuli, is deriving a catalog of post-translational, covalent modifications to the backbone or amino acid side chains of proteins. Most post-translational modifications (PTMs) are added enzymatically, are reversible and dynamic in nature, and thus enable an organism to rapidly respond to environmental changes. Creating such a catalog in UniProtKB requires not only a record of the amino acid change and its position in the sequence but also an understanding of the functional consequences of the presence or absence of this modification.

#### GlyGen

GlyGen (https://www.glygen.org/) is an integrated knowledgebase specifically designed to serve the glycoscience community by providing harmonized and integrated, high-quality data on glycans, glycoproteins and proteins, and glycan-related entities [19]. GlyGen aims to overcome the fragmentation of glycan data across various databases, enabling researchers to explore complex glycan structures, their associated proteins, and their roles in biological processes in one resource [20]. The knowledgebase is a crucial resource for advancing the understanding of glycans, which are essential biomolecules involved in numerous biological functions, including cell–cell communication, immune response, and pathogen recognition. GlyGen serves a distinct role as a cross-domain integration platform that contextualizes protein–glycan relationships. By providing mapped glycan structures and information linked to the proteins, it provides the essential glycomics perspective often omitted in protein-centric resources. This specialized focus enables GlyGen to function as a vital connective resource, harmonizing disparate datasets to ensure that glycan structural information is discoverable and accessible within the broader proteomic and genomic research ecosystems.

At its core, GlyGen is built on the principles of data integration, standardization, and accessibility. The platform integrates data from multiple sources that includes genes, proteins, and glycans from databases such as UniProtKB, NCBI RefSeq [21], PDB [22], GlyConnect [23], GlyCosmos [24], UniCarbKB [25], Ensembl [26], Rhea [27], ChEBI [28], and PubChem [29], ensuring that users have access to comprehensive and curated information. GlyGen’s user interface is designed to be intuitive and accessible, catering to both experienced glycoscientists and those new to the field. The information displayed on the portal always includes evidence details such as the source name, PubMed ID (PMID), and a direct link to the resource from which the data were curated and integrated. This allows users to trace the information back to its original source with ease. The platform also provides a range of tools that allow users to search for specific glycans, proteins, and genes, as well as to explore the relationships between these entities.

In addition to its core data and tool offerings, GlyGen emphasizes the importance of data provenance and quality. Each dataset integrated into GlyGen undergoes rigorous curation and validation processes to ensure accuracy and reliability. The platform also provides detailed metadata and documentation called BioCompute Object (BCO) for each dataset using the IEEE-certified BioCompute specification (https://www.biocomputeobject.org/) [30], allowing users to understand the origin and context of the data they are working with. This commitment to data quality is essential for building trust within the research community and for ensuring that GlyGen can be used as a reliable resource for scientific discovery. GlyGen is also a member of the GlySpace Alliance [31] along with GlyConnect and GlyCosmos, which are other major glycan and glycoconjugate data resources, ensuring the information and data are shared more easily among researchers and databases, and the projects do not duplicate efforts.

#### MatrixDB

The MatrixDB (https://matrixdb.univ-lyon1.fr/) [32] is a member of the IMEx Consortium, which is a group of collaborating databases that curate molecular (mainly protein–protein) interactions with the goal of providing users with a dataset enhanced with controlled vocabulary terms to enable scoring, filtering, and sophisticated searching of molecular interaction information [33]. A detailed curation model has been developed, in which all aspects of an interacting molecule, including PTM status when known, are captured. MatrixDB specializes in the capture of interactions made by proteoglycans, which require a concomitant description of the glycosylation status of the protein(s) that are the participants of the observed interaction, and by glycosaminoglycans, linear polysaccharides consisting of repeating disaccharide units.

#### ChEBI

Chemical Entities of Biological Interest or ChEBI (https://www.ebi.ac.uk/chebi) is a reference database of molecular entities that focuses on the annotation of ‘small’ chemical compounds [28]. Within ChEBI, each chemical entity, including glycans, is assigned a stable and unique ChEBI identifier, ChEBI name, and additional information such as host text-string representation (e.g. WURCS, SMILES, InChI, and InChI key), 2D chemical structure, synonyms, mass, formula, literature evidence, and cross-references to other databases when available [34]. In addition, ChEBI provides an ontology (http://www.obofoundry.org/ontology/chebi.html) to describe the relationships between their chemical entities or entity classes by using a hierarchical taxonomy in which each term (compound or class) can have one or more parent and/or child terms, and describes semantic relationships between these terms to capture chemical structure, function, and classification [35].

## Methods and Materials

### Annotating glycosylated proteins in UniProtKB

Although one of the most common forms of PTM present on human proteins, it is still comparatively unusual to find an unambiguous identification of any single glycan species associated with a specific function played by a particular protein in the experimental literature. The capture of available information has also been hampered, until relatively recently, by the lack of a community-accepted method for describing the content and structure of any set of glycosylations. To meet this challenge, the expert curators at UniProt have devised a series of rules to enable the capture of information currently available in the literature, while actively monitoring both community guidelines and technical advances to ensure that these remain adequate to the information available.

In UniProtKB, glycosylation information is captured at different levels depending on the available evidence. Proteins for which there is evidence that at least one residue is glycosylated are tagged with the keyword ‘Glycoprotein’, enabling users to retrieve and filter glycoproteins by criteria such as species, protein function, or subcellular location. When a protein is known to be glycosylated, but the modified residue has not been identified, this information is recorded in the ‘Post-translational modification’ subsection of the PTM/Processing section as a free-text statement, such as ‘*N*-glycosylated’, together with the supporting evidence. This subsection may also be used to describe the functional consequences of glycosylation when such information is available.

When specific details of glycosylation are known, including the residue, UniProtKB captures this information as a structured site-specific annotation in the ‘PTM/Processing’ Features subsection of the entry (Fig. 1). These annotations use controlled vocabulary terms to describe the feature type, sequence location, modification description, and supporting evidence, allowing relevant entries to be retrieved through targeted search queries. In the text and XML formats of UniProtKB, these position-specific annotations are represented in the feature table (FT) lines.

**Figure 1:**
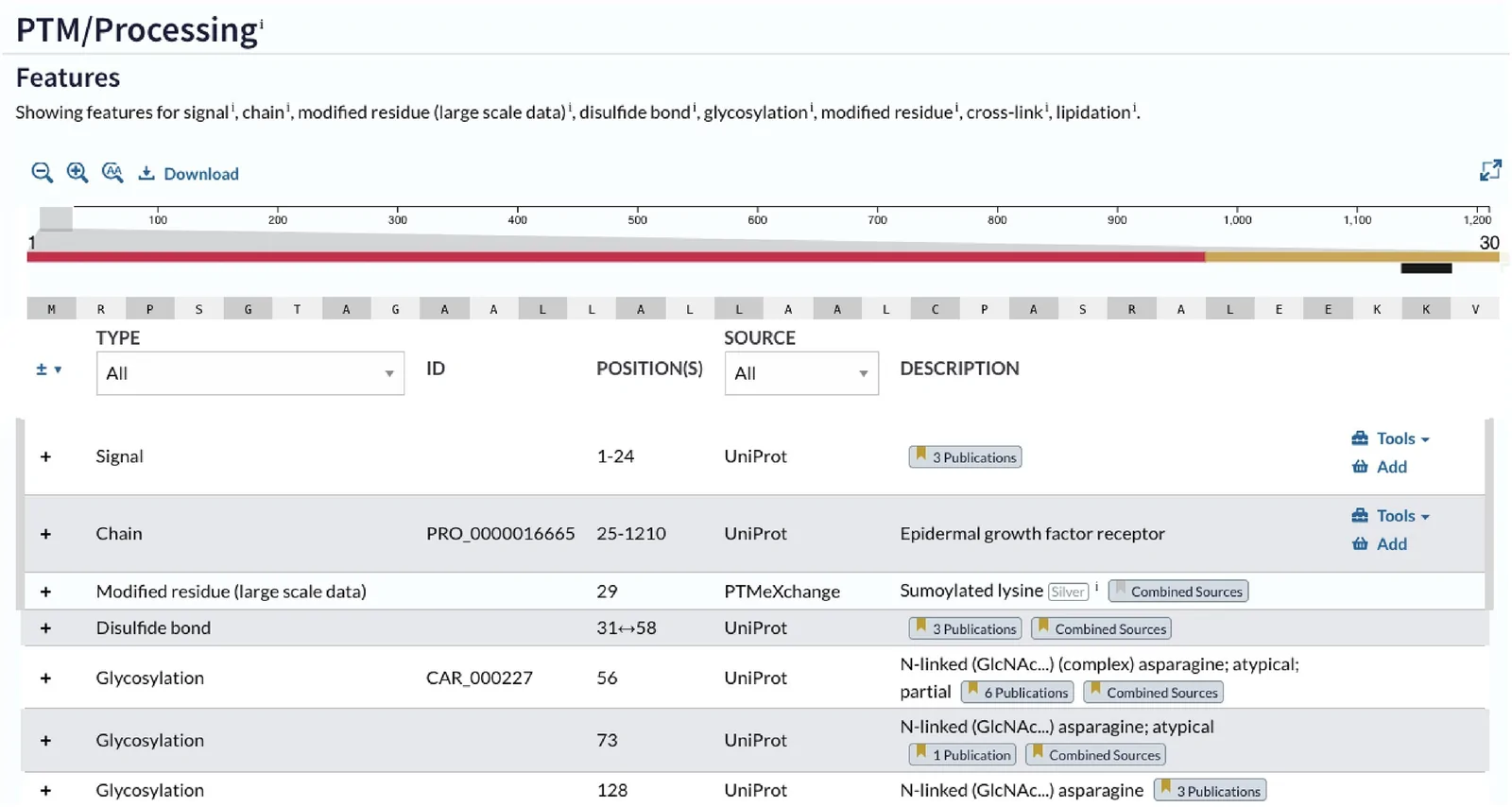
PTM/Processing Features Subsection: View of the PTM/Processing Features Table (Truncated) of the UniProtKB entry for the Epidermal growth factor receptor isoform 1 (P00533-1), showing annotated *N*-linked glycosylation sites (positions 56, 73, 128) with supporting publication counts.

This structured annotation includes the position and type of each covalently attached glycan group (mono-, di-, or polysaccharide), the type of linkage to the protein, and the nature of the reducing terminal sugar (if known) for *N*-glycosylated residues. When the modification is a monosaccharide, the name of the reducing terminal sugar appears alone, whereas for di- and polysaccharides the name is followed by three periods ‘…’ indicating the existence of the additional upstream carbohydrate molecules.

### Glycosylation type-specific annotation

UniProtKB captures five major types of protein glycosylation, each defined by the amino acid residue to which the glycan is attached and the enzymatic or non-enzymatic nature of the reaction (Fig. 2). These are annotated as follows:

- *N*-glycosylation—In eukaryotes, *N*-glycosylation begins as a co-translational event in the endoplasmic reticulum, where preassembled blocks of 14 sugars (2 *N*-acetylglucosamines, 9 mannoses, and 3 glucoses) are added to the nascent polypeptide chain [36]. After cleavage of 3 glucose and 1 mannose residues, the protein is transferred to the Golgi apparatus, where the glycans lose a variable number of mannose residues and acquire a more complex structure during a process called ‘terminal glycosylation’. If there is information pertaining to the structure of the glycan, this will be described in the UniProtKB entry by the controlled vocabulary terms:
  - High mannose (those that have escaped terminal glycosylation)
  - Paucimannose (low mannose content)
  - Hybrid (those with both substituted [GlcNAc linkage] and unsubstituted mannose residues)
  - Complex (with different combinations of mannose, *N*-acetylglucosamine, *N*-acetylgalactosamine, fucose, and sialic acid residues)

**Figure 2:**
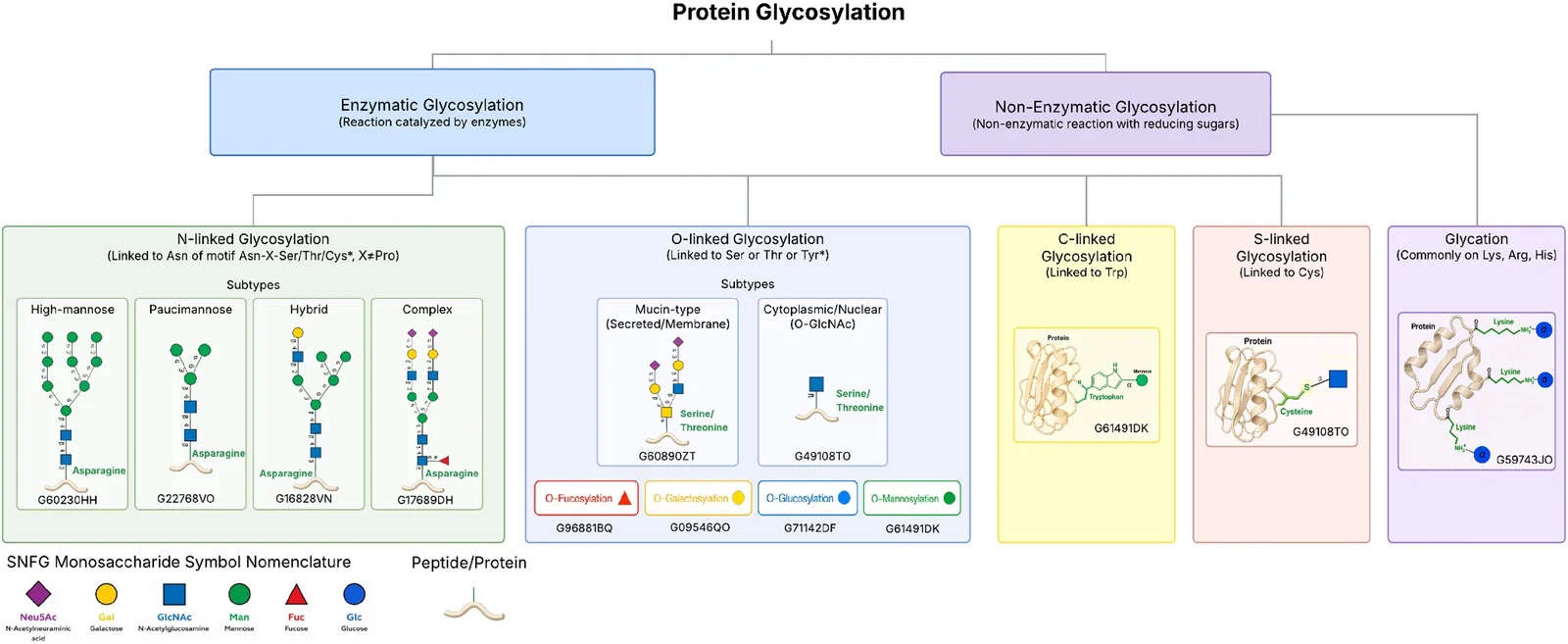
Classification of protein glycosylation types: Overview of major protein glycosylation categories represented in UniProtKB and GlyGen, with representative glycan structures added from GlyGen. Protein glycosylation is broadly divided into enzymatic and non-enzymatic processes. Enzymatic glycosylation includes *N*-linked glycosylation, which involves attachment of glycans to the asparagine (Asn) side chain within the consensus sequon Asn-*X*-Ser/Thr/Cys (where *X* is any amino acid except proline) and is subdivided into four structural classes: high-mannose, paucimannose, hybrid, and complex. *O*-linked glycosylation occurs at the hydroxyl groups of serine (Ser), threonine (Thr), or, less commonly, tyrosine (Tyr) residues, and includes mucin-type glycans found on secreted and membrane proteins as well as cytoplasmic/nuclear *O*-GlcNAc modifications. Additional *O*-linked variants include *O*-fucosylation, *O*-galactosylation, *O*-glucosylation, and *O*-mannosylation. Less common *C*-linked and *S*-linked glycosylation are represented by mannose attached to tryptophan (Trp) and GlcNAc attached to cysteine (Cys), respectively. Non-enzymatic glycosylation, or glycation, results from the spontaneous reaction of reducing sugars (such as glucose) with primary amine groups, predominantly on lysine (Lys) residues, without enzymatic catalysis. Representative glycan structures for each category are shown using the Symbol Nomenclature for Glycans (SNFG), with corresponding GlyTouCan accession identifiers listed beneath each structure. Monosaccharide symbols: purple diamond, *N*-acetylneuraminic acid (Neu5Ac); yellow circle, galactose (Gal); blue square, *N*-acetylglucosamine (GlcNAc); green circle, mannose (Man); red triangle, fucose (Fuc); blue circle, glucose (Glc). For *N*- and *O*-linked glycosylation examples, the glycan attachment site is represented by a simplified peptide/protein backbone rather than a full protein structure. Asterisks denote less common glycosylation residues.

UniProt curators will capture both experimentally verified *N*-glycosylation sites and those identified by an approved list of predictive tools, but these sites are generally not propagated to orthologs by annotation transfer.

- *O*-glycosylation—*O*-linked glycosylation of secreted and membrane-bound proteins is a post-translational event that takes place in the *cis*-Golgi compartment after *N*-glycosylation and folding of the protein. It refers to the attachment of glycans to serine and threonine, and, to a lesser extent, to hydroxyproline and hydroxylysine, with sugars being added in a stepwise fashion. The most common type of *O*-glycosylation in secreted and membrane-bound mammalian proteins seems to be the addition of reducing terminal *N*-acetylgalactosamine (GalNAc)—mucin-type glycosylation—which can be further extended with galactose (Gal), *N*-acetylglucosamine (GlcNAc) or GlcNAc and Gal, resulting in eight common core structures, which are often further decorated with the addition of up to three sialic acid residues. *O*-linked glycans play important roles in protein localization and trafficking, protein solubility, antigenicity, and cell–cell interactions. Some cytoplasmic and nuclear proteins have simple *O*-linked glycans in which a single *N*-acetylglucosamine residue is linked to a serine, threonine, or tyrosine. Predicted sites are only annotated in UniProtKB in regions of proteins that are known or predicted to be extracellular, or are described in cases where mucin-type *O*-glycosylation has been observed, but the exact site is unknown.
- *C*-linked glycosylation—*C*-linked glycosylation refers to the covalent attachment of a mannose residue to a tryptophan residue within the polypeptide chain of an extracellular protein. This is only captured by UniProt curators when experimental evidence is available.
- *S*-linked glycosylation refers to the attachment of oligosaccharides to the sulfur atom of the cysteine. This modification seems to be extremely rare in comparison to other glycosylation events and was first seen in human and bacterial peptides. This is only captured by UniProt curators when experimental evidence is available.
- Glycation—the Maillard reaction is the irreversible, non-enzymatic binding of glucose with the nitrogen atoms (*N*-terminus, lysine, and histidine side chains) of proteins and also to lipids. Glycation is associated with diabetes and the aging process. This is only captured by UniProt curators when experimental evidence is available.

While the position and type of the glycosylation site are recorded in the entry, actual details of the glycan chain or chains are not. A free text field (PTM) allows the curator to record more specific details and any comments they may wish to make on the effect of the glycosylation on protein function, and cross-references to resources such as GlyGen enable the user to access more information about the glycoconjugate.

### Annotating glycosyltransferases and glycohydrolases in UniProtKB

When active enzymes are expertly curated in UniProtKB, enzyme-specific information is added describing the catalytic activity, the regulation mechanism, whether a cofactor is required, and the positions of active site(s), cofactor, and substrate binding sites. The catalytic activity is defined both by the Enzyme Commission classification system [37] and by a structured description of the biochemical reaction the entry catalyzes provided by the Rhea database [38]. When the enzyme is active as a multi-subunit complex, this is described both in free text in the CC INTERACTION section and, more systematically, in collaboration with the Complex Portal [39]. Glycosyltransferases are classified into families, as defined by the CAZy database (http://www.cazy.org), which describes the families of structurally related catalytic and carbohydrate-binding modules (or functional domains) of enzymes that degrade, modify, or create glycosidic bonds [40].

### Annotating glycosylated proteins in IMEx Consortium databases

The IMEx curation rules describe the role of any PTM term using the HUPO PSI-MI CV as either an ‘observed-ptm’ (MI:0925), where there is no evidence of whether it is required for the interaction or not, a ‘prerequisite-ptm’ (MI:0638), which is required for an interaction to occur, or as a ‘resulting-ptm’ (MI:0639), i.e., an event such as a cleavage which is the result of an interaction. Observed PTMs can be described in more detail as either decreasing (MI:1223), disrupting (MI:1225), or increasing (MI:1224) the interaction. The PTM itself is described using HUPO PSI-MOD CV, either the parent term ‘glycosylated residue’ (MOD:00693) or a child term. *N*-glycosylated (MOD:00006), *O*-glycosylated (MOD:00396), *C*-glycosylated (MOD:00421), and *S*-glycosylation (MOD:00 426)—each of these has a number of child terms enabling a more detailed description of the glycosylation, such as N4-glycosyl-L-asparagine (MOD:00160). PTMs are linked to either the specific residue in the amino acid sequence of a protein, which is known to be glycosylated, or as an ‘undetermined sequence position’ (MI:0339) if the exact position is unknown.

### Annotating glycosaminoglycans in IMEx Consortium databases

Glycans (CHEBI:167559) such as heparin (CHEBI:28304) are identified as participants in an interaction using ChEBI identifiers, taking advantage of the fact that the ChEBI database is also organized as an ontology with ‘glucosaminoglycan’ (CHEBI:49236) as a parent term enabling a more granular description of the molecule if known, for example *N*(4)-{α-d-mannosyl-(1→6)-[α-d-mannosyl-(1→2)-α-d-mannosyl-(1→2)-α-d-mannosyl-(1→3)]-β-d-mannosyl-(1→4)-*N*-acetyl-β-d-glucosaminyl-(1→4)-*N*-acetyl-β-d-glucosaminyl}-l-asparagine (CHEBI:52269). The number of repeating units in the chain can be captured as a structured annotation comment using a controlled syntax under the heading ‘polymer chain length’ (MI:2278).

### Annotating glycans in ChEBI

To help identify glycans in their database, ChEBI includes numerous ontology terms for glycan molecules organized into a hierarchy. The most general term is ‘carbohydrates and carbohydrate derivatives’ (CHEBI:78616), which serves as the parent of more specific terms such as carbohydrate (CHEBI:16646), polysaccharide (CHEBI:18154), oligosaccharide (CHEBI:50699), and glycan (CHEBI:167559). These terms can themselves be parents of more precise classifications, such as ‘amino tetrasaccharide’ (CHEBI:59412) or acetamides (CHEBI:22160). Compounds are also classified based on chemical structure and biological roles within the ChEBI ontology, placing carbohydrate structures into a hierarchy through relational statements such as ‘β-d-glucose is_a d-glucose, which is_a d-aldohexose, which is_a monosaccharide, which is_a carbohydrate’ [34, 41]. Annotating glycans in ChEBI also involves standardizing their structures, linking them to external databases, providing biological context, and adding literature references. Glycan structures are curated using standardized notations such as IUPAC, GlycoCT, and WURCS, ensuring an accurate representation of monosaccharide composition, linkages, and anomeric configurations. Using these annotation standards, approximately 29,328 ChEBI entries were identified as glycans based on both cross-reference and ontology-based criteria. Entries were considered glycans if they contained a cross-reference to GlyTouCan, GlyGen, KEGG-Glycan, or a PubChem compound associated with a GlyTouCan ID, or if their WURCS or IUPAC sequence could be mapped to a GlyTouCan ID using the GlyLookup tool. In addition, included entries were required to be classified under the ChEBI term ‘carbohydrates and carbohydrate derivatives’ or one of its child terms.

### GlyGen curation of glycosyltransferase and glycohydrolase data from UniProtKB

When a new species is to be added to the next GlyGen release, glycosyltransferase (GT) and glycohydrolase (GH) enzymes for this species are retrieved from the UniProtKB database, then manually checked, curated, and formatted so that they can be integrated into GlyGen. GT and GH enzymes are retrieved using the UniProtKB advanced search interface, in which the database search is restricted to reviewed entries that have the NCBI taxonomic identifier for the new species and contain either the keyword ‘Glycosyltransferase [KW-0328]’ for the GT dataset or the keyword ‘Glycosidase [KW-0326]’ for the GH dataset. Once a search has been performed, the data displayed in the subsequent results page are then viewed as a table and customized using the ‘Customize Column’ option so that the GH or GT results contain the following data: ‘Entry’, ‘Organism’, ‘Reviewed’, ‘Gene Names’, ‘Protein Names’, ‘EC Number’, ‘BRENDA’, ‘CAZy’, ‘InterPro’, ‘Pfam’, and ‘Gene Ontology (molecular function)’. The customized results table is then downloaded as a tsv file. The downloaded datasets are then manually checked to ensure that there is strong evidence for their function as a GH or GT enzyme, e.g., their EC number and CAZy family ID are correct, and/or there is experimental evidence in papers associated with the enzyme. The datasets are then standardized and formatted so that they can be integrated into GlyGen. The dataset and its BCO are available for download at data.glygen.org (e.g. https://data.glygen.org/GLY_000025).

## Results

### Annotated glycosylated proteins in UniProtKB

To date (release 2026_01), 33 401 proteins have been manually annotated as glycoproteins in UniProtKB, with a further 5 790 601 unreviewed entries having this annotation added by automatic annotation transfer or predictive tools. A total of 32 186 reviewed proteins have one or more sites mapped to the sequence level; this is only true of 83 189 of the unreviewed entries. *N*-glycosylation is the most frequently annotated type found in 31 017 reviewed entries; however, only 3068 of those entries are tagged as having experimental evidence for any of the glycosylation sites. Only 1944 reviewed *O*-glycosylated entries, 534 with experimental evidence. Only 74 reviewed entries are *C*-glycosylated, 13 *S*-glycosylated, and 26 glycated. The ‘evidence’ is described by a term from the Evidence and Conclusion Ontology (ECO) [42].

### The UniProtKB website view

The UniProt website (www.uniprot.org) provides access to UniProtKB protein sequence records and annotations, including carbohydrate binding site annotations for glycosylated proteins. Using the ‘Advanced Search’ option (Fig. 3A), it is possible to search for proteins mapped at the sequence level to specific types of glycosylation and distinguish between those confirmed by experimental evidence and those onto which the annotations have been transferred by sequence similarity. The resulting search page displays the matching UniProtKB entries in a table (Fig. 3B), together with key information such as accession number, entry name, protein name, gene name, organism, and sequence length. The sidebar facets can be used to further refine the results by review status, organism, taxonomy, protein features, or other criteria relevant to the user’s research question. For an individual entry, users can then open the UniProtKB record and view detailed glycosylation annotations in the PTM/Processing section, where the modified residue, glycosylation type, sequence position, and supporting evidence are displayed. This enables the user to understand, for example, the potential effect of a disease-related amino acid variant if it mutates out a glycosylation site essential for protein function.

**Figure 3:**
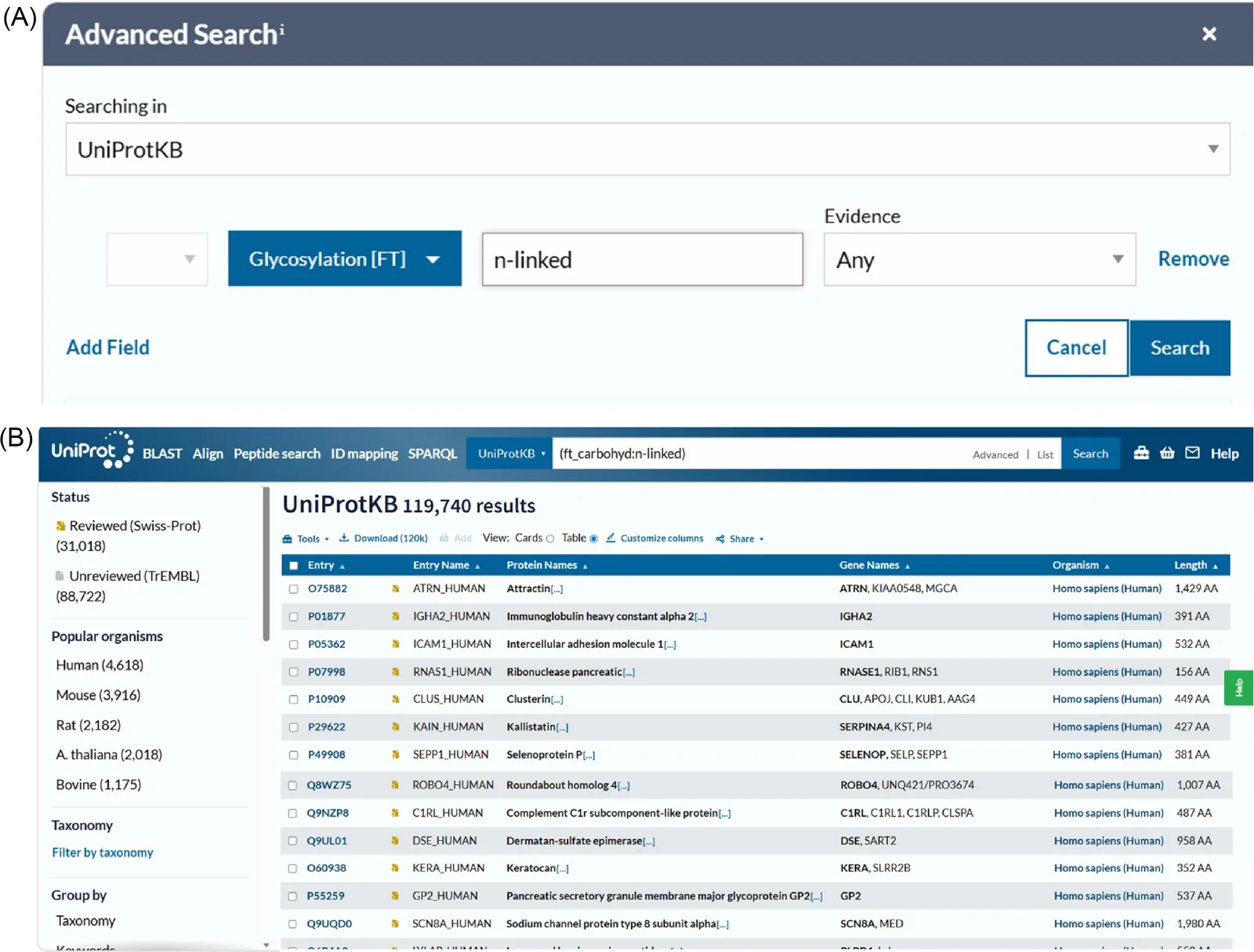
UniProtKB Advanced Search and results for *N*-linked glycosylated proteins. (A) The Advanced Search interface in UniProtKB is configured to retrieve entries with the ‘Glycosylation (FT)’ feature set to ‘n-linked’. The evidence filter is set to ‘Any’. (B) The UniProtKB results page shows entries retrieved using the query for *N*-linked carbohydrate features, with filtering options for reviewed and unreviewed entries, organism, taxonomy, and protein features.

### Annotated glycosylated proteins and proteoglycans in the IMEx data resources

The IMEx data model enables data resources to select whether to access/download some or all of the data. For example, MatrixDB only reuses data annotated by any consortium member that is relevant to extracellular matrix proteins, whereas the IntAct database shows all data and enables the user to filter by their area of interest. The Advanced Search option created by the IntAct database enables searching by protein features such as PTM, so it is possible to find, for example, all proteins involved in an interaction that have been observed to be *n*-glycosylated (MOD:00006) (Fig. 4).

**Figure 4:**
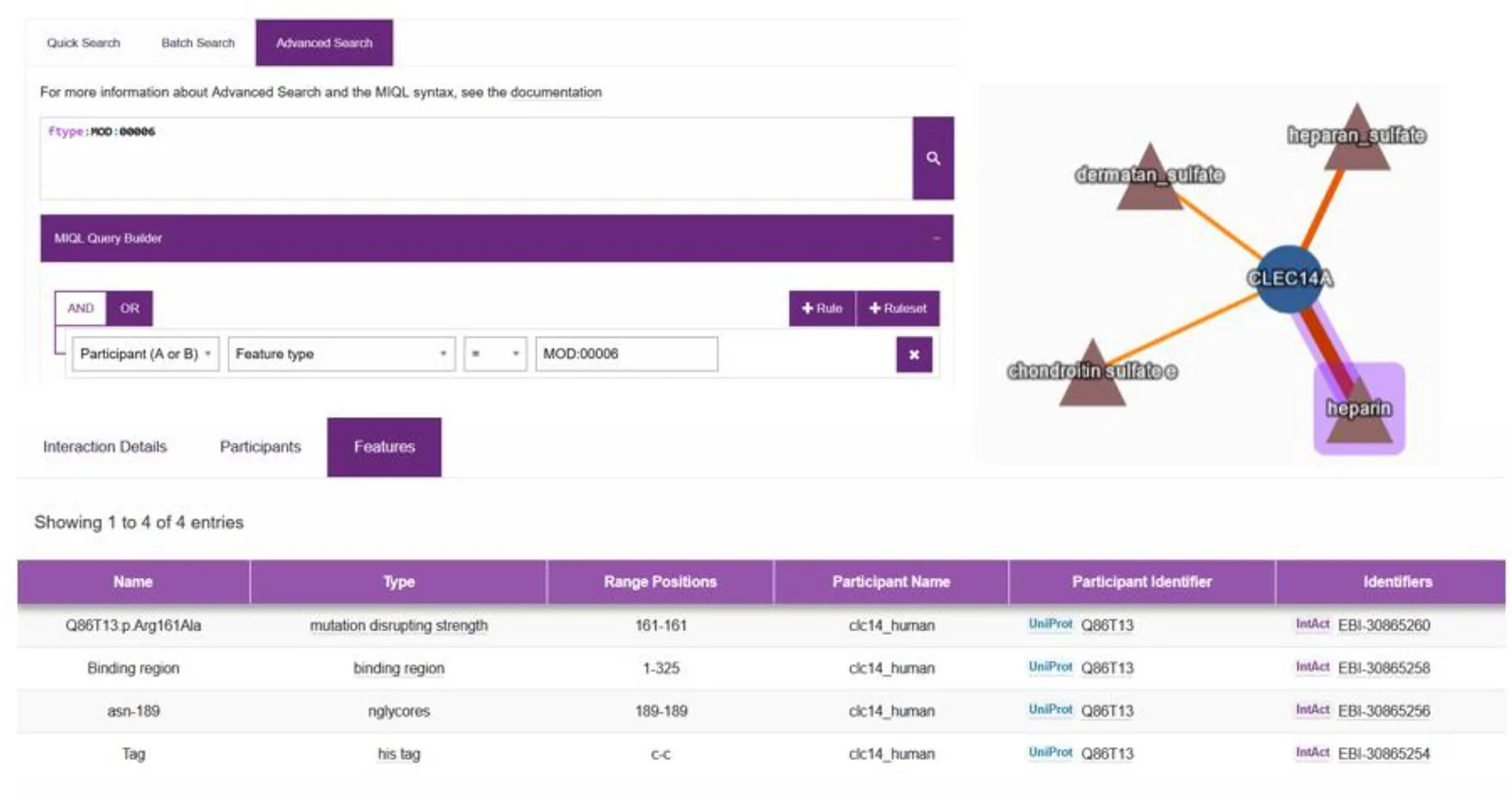
Using IntAct’s advanced search to identify proteins annotated with the *N*-glycosylation modification term (MOD:00006): Example results for CLEC14A (Q86T13) show an annotated *N*-glycosylation site (Asn189), a mutation affecting interaction strength (Arg161Ala), and binding regions. The network view depicts CLEC14A interactions with glycosaminoglycans, including dermatan sulfate, heparan sulfate, chondroitin sulfate, and heparin.

The complete ChEBI ontology is indexed, so that searches on the IMEx website (www.imexconsortium.org) using identifiers for higher-level grouping classes in the ChEBI ontology will retrieve UniProtKB records with binding site annotations to all child classes. For example, searching with the identifier CHEBI:18085 (glycosaminoglycan) will bring down all interactions annotated to this class of molecule, although the actual annotations will be made more specifically to entities such as heparin or hyaluronan (CHEBI:16336). Searching the IntAct website for interactions made by heparin enables further filtering, for example, by interaction confidence, species, or interactor type and will allow highlighting of those interactions known to be affected by amino acid mutations in the target proteins (Fig. 5). A tabular view gives access to more information. In MatrixDB, all 62 glycosaminoglycans or GAGs entries contain a link to ChEBI, the glycan structure in both 3D and the SNFG format, the GlycoCT digital sequence, and, when available, a link to the corresponding entry in the GlyTouCan database.

**Figure 5:**
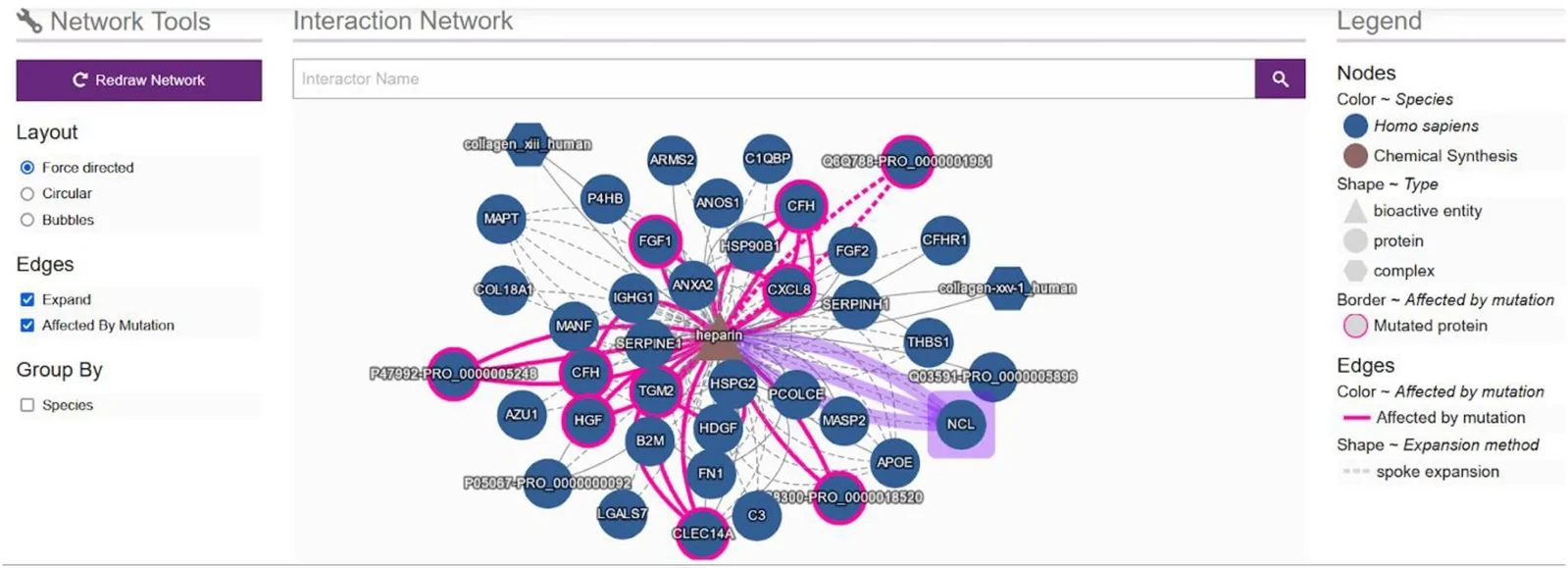
IntAct visualization of interactions between heparin (CHEBI:16336) and multiple *Homo sapiens* proteins: Nodes are color-coded by species (dark blue: human, brown: chemical entities), with shapes indicating molecular type (circles: proteins, hexagons: chemicals, rectangles: complexes). Pink-bordered nodes indicate proteins affected by mutation; magenta edges highlight interactions altered by mutation.

### Annotated glycans in ChEBI

Cross-references are added to glycans in GlyGen and their corresponding ChEBI entities, using both automatic and manual procedures. Cross-references are automatically generated using the database_accession.tsv file from the ChEBI-EBI FTP (https://ftp.ebi.ac.uk/pub/databases/chebi/Flat_file_tab_delimited/) as the mapping file. GlyTouCan accessions of the glycans are mapped to the CHEBI ID through their PubChem Compound ID (CID) (if the PubChem CID has a ChEBI cross-reference), or if glycans with a PubChem CID lack a ChEBI cross-reference, glycans are integrated into ChEBI using applications like KNIME [43] and ClassyFire [44].

If glycans lack a PubChem CID, they are manually submitted to the ChEBI database using their submission tool. GlyGen and GlyTouCan cross-references are added as ‘Manual X-ref’ to the individual glycan ChEBI entries, and their ChEBI identifiers are provided in the corresponding GlyGen entry for cross-referencing [34]. In addition, ambiguously defined glycans not currently in ChEBI are submitted to the database for integration by GlyGen using the ChEBI Submission Tool. The submitted glycans are annotated with information such as name (which is the source database GlyTouCan and the GlyTouCan ID, e.g. ‘GlyTouCan G06110VR’), monosaccharide composition, digital sequence (WURCS sequence), and ontology class. The ontology classes of the ambiguously defined glycans are based on the Glycan Naming ontology or GNOme (https://obofoundry.org/ontology/gno, https://gnome.glyomics.org/) and include classes such as base composition (https://www.ebi.ac.uk/chebi/searchId.do?chebiId=CHEBI:167481), composition (https://www.ebi.ac.uk/chebi/searchId.do?chebiId=CHEBI:167502), and topology (https://www.ebi.ac.uk/chebi/searchId.do?chebiId=CHEBI:167503) within the ChEBI ontology that is linked to the corresponding terms in GNOme [34]. Entries are also manually curated on request from submitters, which include other databases such as Rhea, UniProtKB, and members of the IMEx Consortium, primarily MatrixDB. Submitted glycans are immediately searchable and visible on the ChEBI website as 2-star (unchecked) entries, and are upgraded to 3-star (checked) status once they have been checked by a ChEBI curator [34].

GlyGen is currently working with ChEBI to use their automated FTP-based bulk submission option to submit all GlyGen glycans to ChEBI at each GlyGen release. This will dramatically increase coverage of glycans in ChEBI and the cross-references between the two resources. It will also enable us to keep the GlyGen glycans in ChEBI up to date and consistent with their corresponding glycan in GlyGen.

### Integration and exchange of data between GlyGen, UniProtKB, and other resources

The data curation workflow and bidirectional exchange between UniProtKB, GlyGen, and other glycoscience resources are summarized in Fig. 6. UniProtKB provides protein and protein-related information for all proteins in the Reference Proteome of a given NCBI taxonomy ID for 16 organisms currently integrated in the GlyGen database. For each protein, basic information such as gene-centric canonical and isoform mapping, cross-references to various databases, annotations, citations, keywords and GO classification, protein sequence, PTM annotations including glycosylation and phosphorylation, enzyme information, ligands, Rhea catalytic activity, Reactome pathway and reaction annotations, genomic coordinate information, such as Ensembl gene ID, transcript ID, chromosome, and start and end positions, is provided and packaged in RDF N-Triples (RDF/NT) format, a machine-readable format that supports standardized data exchange, integration, and linking across biological resources [20].

**Figure 6:**
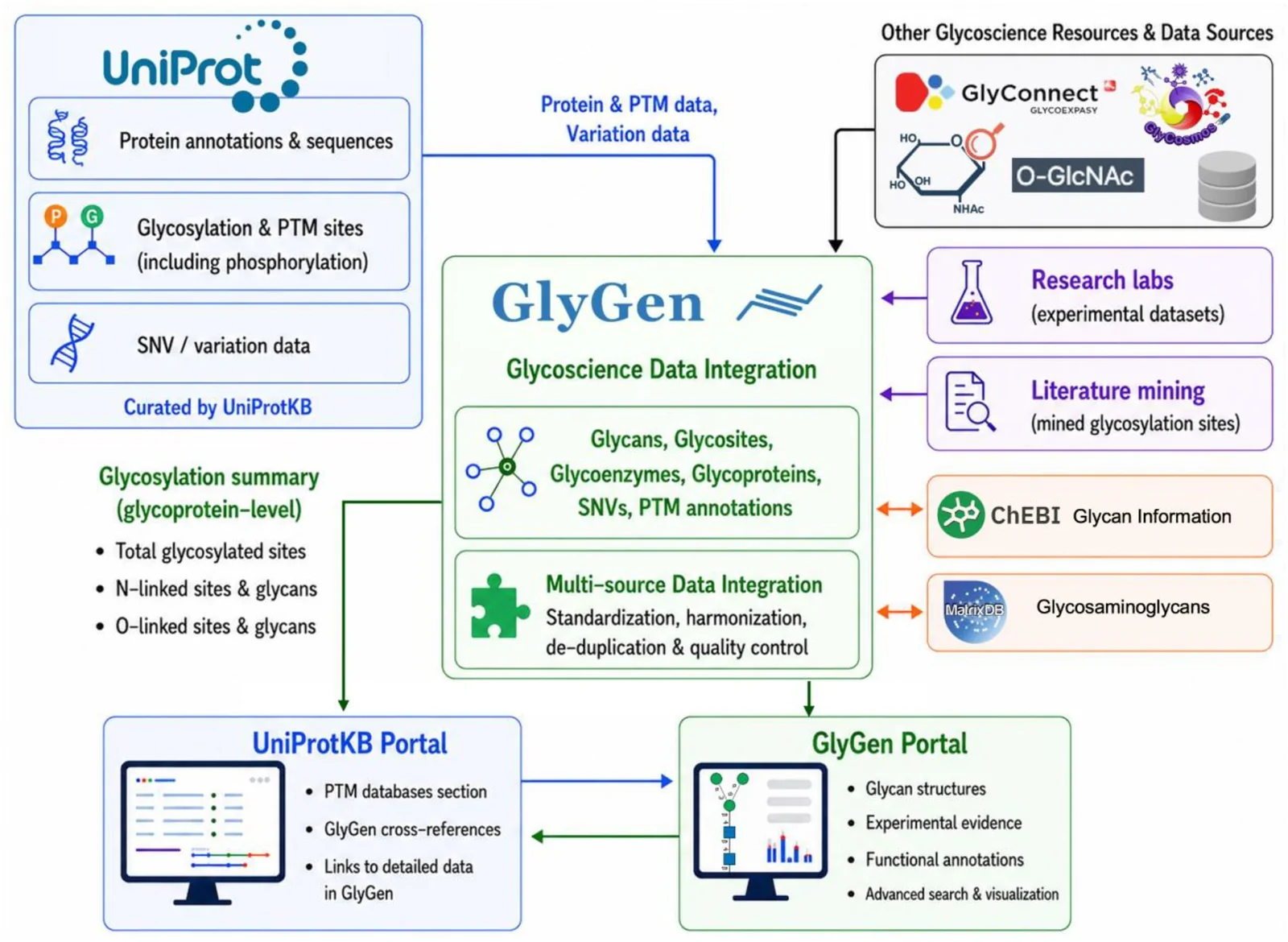
Data flow between UniProtKB, GlyGen, and other glycoscience resources: Schematic overview of the curation workflow and data exchange between UniProtKB and GlyGen. UniProtKB provides manually curated protein annotations, glycosylation and PTM sites, and variation data to GlyGen. GlyGen further integrates glycan and glycosylation information from other glycoscience resources such as GlyConnect, GlyCosmos, and O-GlcNAc Database, including ChEBI and MatrixDB, experimental datasets from research laboratories, and literature-mined sites. In exchange, GlyGen provides UniProtKB with a glycoprotein-level glycosylation summary that is displayed on the UniProtKB Portal with cross-references linking users to detailed glycan-level information on the GlyGen Portal.

In addition, UniProtKB also provides GlyGen with somatic and germline single-nucleotide variation (SNV) data from the large-scale initiatives like dbSNP, 1000 Genomes, ExAC, ClinVar, TCGA, COSMIC, TOPMed, gnomAD, PeptideAtlas, MaxQB, EPD, ProteomicsDB, and HPA. The data also contain gene-to-protein mappings, reference and alternative alleles, minor allele frequency, mutation type, PolyPhen score, prediction score, disease description, cross-references, and publication evidence. Analyzing variation site data in conjunction with glycosylation site data can reveal critical insights into how genetic variations may affect glycosylation patterns. Specifically, this approach can identify sites where glycosylation might be lost or gained due to mutations. For example, certain missense mutations can alter glycosylation motifs, potentially leading to a loss of glycan attachment at specific sites, which can affect protein folding, stability, and function. Conversely, mutations that create new glycosylation motifs could result in the gain of glycosylation, which may impact protein interactions and localization. This integrative analysis is crucial for understanding the molecular mechanisms underlying diseases linked to glycosylation abnormalities, such as congenital disorders of glycosylation and certain types of cancer.

The datasets, packaged and released for GlyGen, follow the UniProt release cycle. Data are usually made available within a week of UniProt release. The latest release is 2025_02, available at https://ftp.ebi.ac.uk/pub/contrib/glygen/current_release/. These data are combined with glycan and other data from other repositories to make a GlyGen release that powers the GlyGen backend and ultimately the frontend portal.

GlyGen integrates protein annotations and glycosylation site information curated by UniProtKB; however, UniProtKB is not the sole source of glycosylation data in GlyGen. GlyGen also integrates glycosylation information from other specialized resources, including GlyCosmos, GlyConnect, and the O-GlcNAc Database. Beyond these curated resources, GlyGen incorporates glycosylation sites derived directly from experimental data generated by research laboratories, as well as sites identified through literature mining tools. While GlyGen’s mission is to serve as a comprehensive glycoscience resource with broad coverage of glycosylation sites, it does not aim to perform manual curation of glycosylation data from the primary literature in the manner of UniProtKB or similar resources. To further strengthen this relationship, GlyGen proposes the inclusion of new high-impact publications on glycosylation in UniProtKB, ensuring that the latest research findings are integrated into the database, and actively monitors data consistency, promptly alerting UniProtKB to any discrepancies to enhance overall data accuracy and reliability.

In exchange, GlyGen provides UniProtKB with a specialized dataset that contains information on these additional glycosylation sites. This dataset includes key information such as the UniProtKB protein accession number, the total number of glycosylated sites on each glycoprotein, and a breakdown of these sites into *N*-linked and *O*-linked glycosylation. For each glycoprotein, the dataset specifies the total number of *N*-linked sites along with the distinct *N*-glycans identified at those sites, as well as the total number of *O*-linked sites with their corresponding distinct *O*-glycans. This summarized glycosylation data are accessible on the UniProtKB portal in the ‘PTM databases’ subsection, which is located under the ‘PTM/Processing’ section of each glycoprotein entry page (Fig. 7). The UniProtKB protein accession numbers are hyperlinked directly to GlyGen’s glycosylation section for that glycoprotein, allowing users to explore more detailed information, such as specific glycan structures, functional annotations, and experimental evidence. For example, in the PTM/Processing section for EGFR, UniProtKB annotates 13 experimentally supported *N*-linked glycosylation sites (Fig. 1). Users can also follow the GlyGen cross-reference in the PTM section of EGFR to explore a broader dataset of 25 glycosylation sites with identified glycans, comprising 71 *N*-linked glycans across 17 sites and 1 *O*-linked glycan across 8 sites, with an additional 82 glycans lacking precise site assignment (Fig. 7). This integration enhances the utility of both resources, providing researchers with comprehensive insights into protein glycosylation.

**Figure 7:**
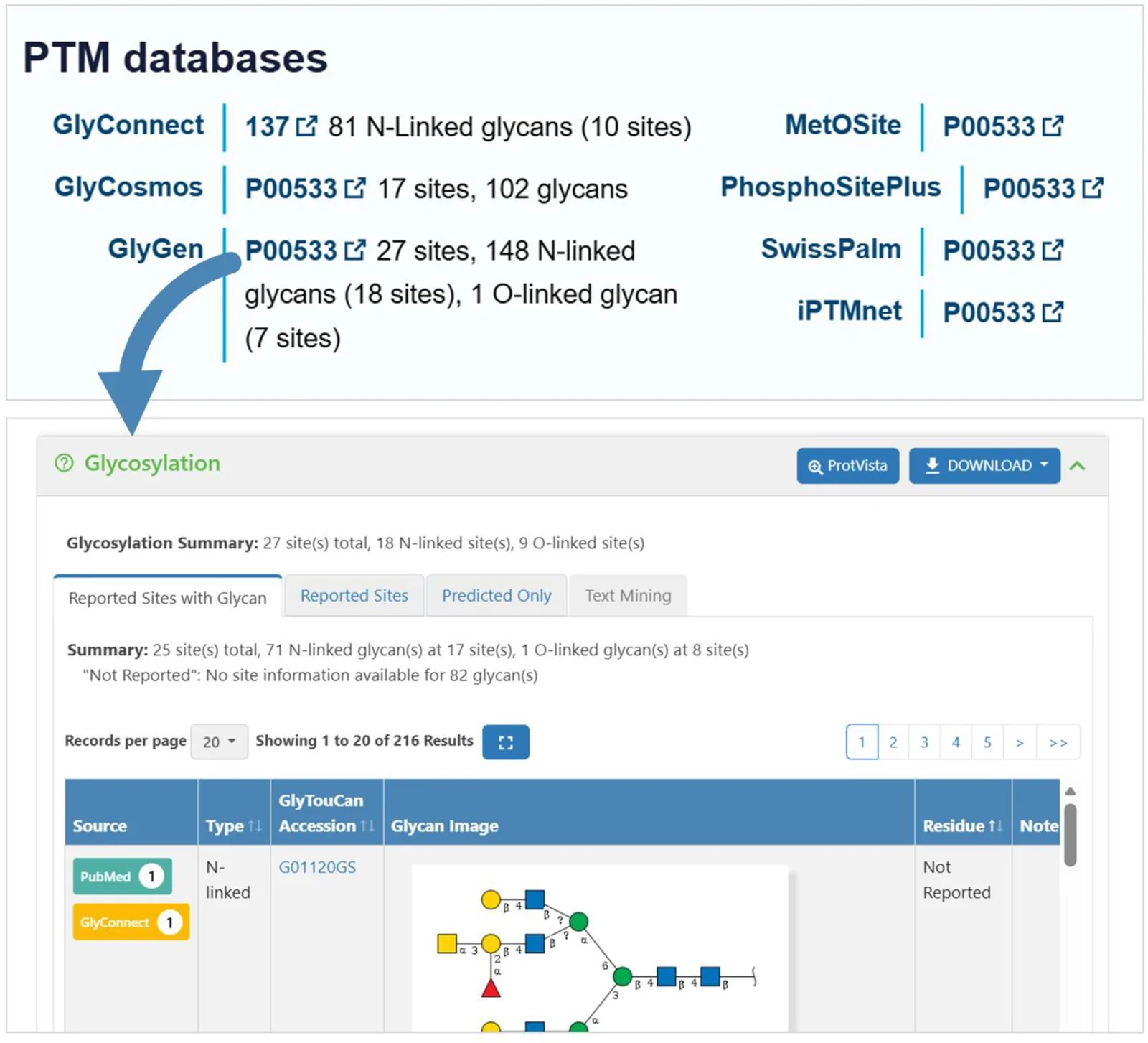
PTM databases subsection: PTM databases subsection of the UniProtKB entry for EGFR (P00533) showing a GlyGen cross-reference and a summary of the number of different linkage types and sites of the 25 reported glycosylation sites with glycans in GlyGen. The link directs users to the EGFR glycosylation table in GlyGen, which provides detailed information on glycan structures, glycosylation sites, and evidence sources. Note that any discrepancies between the glycosylation summary shown in the GlyGen cross-reference within UniProtKB and the summary at the top of the GlyGen glycosylation table reflect differences in the UniProt and GlyGen release schedules.

GlyGen integrates glycomic data from ChEBI and glycomic and proteomic data from MatrixDB, mapping this information to the corresponding glycan and protein pages while providing cross-references back to the original ChEBI or MatrixDB entries.

For ChEBI, GlyGen currently includes cross-references in 9910 glycans, generated by mapping data from the ChEBI direct download files (database_accession.tsv), available via the ChEBI-EBI FTP server (ftp://ftp.ebi.ac.uk/pub/databases/chebi/), to their associated GlyTouCan accessions in GlyGen. The ChEBI cross-reference dataset and its BCO, including details on how the dataset was generated, are available for download at https://data.glygen.org/GLY_000296. ChEBI also links back to GlyGen, with 29,328 glycan entries containing GlyGen cross-references.

For MatrixDB, GlyGen generates interaction datasets for proteins and glycosaminoglycans (GAGs) from human, mouse, and rat data (e.g. https://data.glygen.org/GLY_000332 and https://data.glygen.org/GLY_000338), adding MatrixDB cross-references to both the 271 proteins and 13 glycan interactors in the GlyGen database.

### GlyGen isoform mapper

Each UniProtKB/Swiss-Prot entry contains all curated protein products encoded by a given gene in a given species or strain. For each entry, a canonical sequence is chosen for display, based on whether it is functional, widely expressed, encoded by conserved exons found in orthologous sequences, identical to consensus sequences chosen by other resources and genome curation efforts such as CCDS and MANE or is the longest sequence. The canonical sequence generally allows for the description of the majority of functionally important domains, motifs, sites, and post-translational modifications, naturally occurring variants with functional and clinical significance, as well as other sequence features (https://www.uniprot.org/help/canonical_and_isoforms).

The GlyGen Isoform Mapper tool allows for finding and mapping the site position and amino acid of a UniProtKB isoform protein sequence to the corresponding UniProtKB canonical isoform protein sequence. The tool plays a crucial role in facilitating the transfer of annotations from the original isoform to the canonical isoform, thereby preventing the loss of information when annotations are integrated exclusively for the canonical isoform proteins by the bioinformatics resources. For a given isoform accession, the GlyGen Isoform Mapper tool finds the corresponding canonical gene-centric grouped accession by using the BioJava Alignment Module https://biojava.org/. It uses the Global alignment - Needleman and Wunsch algorithm to find the amino acid index on the canonical sequence from the isoform sequence. To allow easy access and to allow different input data formats, GlyGen has provided three different input interfaces to the user: Accession- and Site-based, File-based, and Sequence- and Site-based. Accession- and File-based interfaces use UniProtKB accession for mapping, and the Sequence-based interface in GlyGen first internally retrieves the UniProtKB accession from the protein sequence before proceeding with mapping. The tool specifically targets all the available UniProtKB accessions present in GlyGen. UniProtKB accession or protein sequence and amino acid position are mandatory inputs, and amino acid is an optional user input. Users can add new data, use existing examples, edit tables, copy data using the clipboard, and upload a file to enter the data. All three interfaces currently limit user input to 1000 rows; this limit will be revised in the future based on feature requirements and user feedback. The interface provides data validation messages to the user before proceeding. Once a user proceeds with the request, mapping is done, and the user is provided with the result page. The results page displays user input, the actual amino acid, the corresponding mapped canonical accession, the amino acid position, and the amino acid. A warning is provided in case the input amino acid is incorrect, and an error is provided in case there is a failure in mapping due to the input accession or the input amino acid position. On the results page, a user can download the results in multiple file formats, use the option to modify input data, or explore a protein by visiting the specific protein details page (Fig. 8).

**Figure 8:**
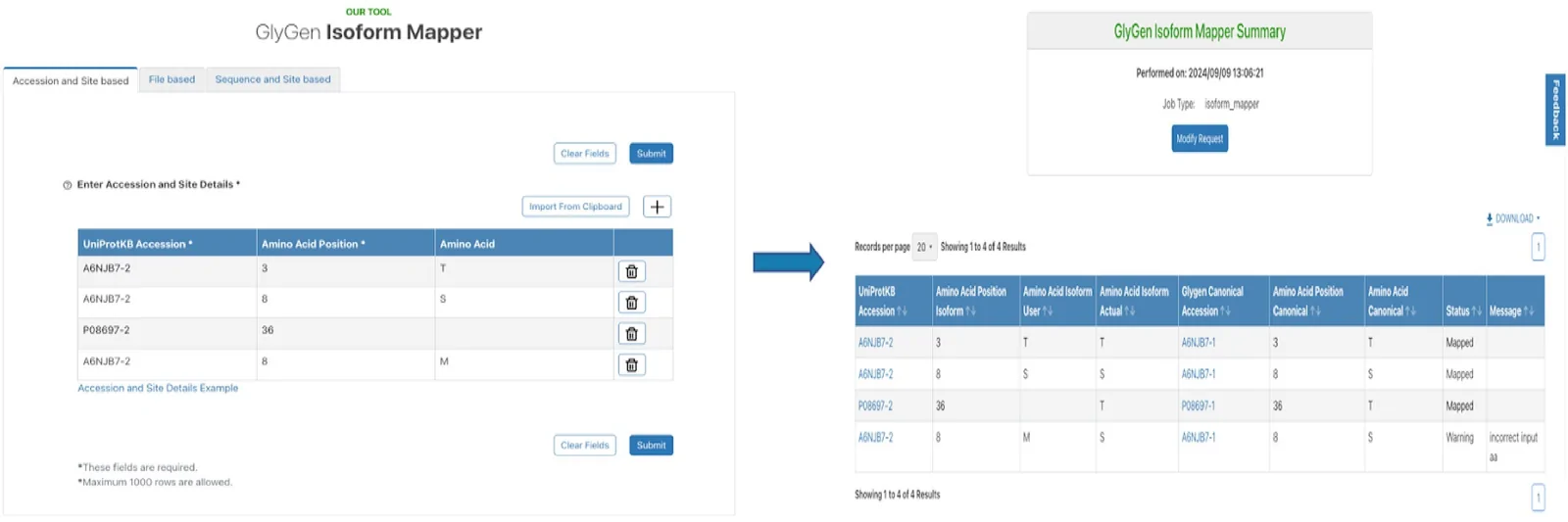
GlyGen Isoform Mapper Tool: User input interface of the Isoform Mapper Tool (left) showing UniProtKB isoform accessions, amino acid positions, and residues. The output summary (right) maps isoform positions to canonical accessions using global sequence alignment. Results indicate the mapped position, amino acid identity, and status (e.g. ‘Mapped,’ ‘Incorrect input aa’).

### ProtVista for glycosylation sites

UniProt has developed ProtVista as part of the Nightingale library of reusable data visualization web components [45], which enables the visualization of protein features such as PTMs in the context of the protein sequence and other features such as amino acid variants, protein domains, and 3D structure.

Visualizing functional protein residues and structural characteristics in the context of glycosylation sites offers a powerful approach for exploring and interpreting glycosylation within biological systems. To support this exploration, GlyGen has integrated the UniProt ProtVista protein visualization browser, providing a comprehensive representation and access to the functional amino acid residue annotations and to the glycan and glycosylation site annotations. The integration of ProtVista into GlyGen addresses a critical gap in existing resources: the need to visualize glycosylation sites not in isolation, but in the direct context of protein sequence and annotations. While UniProt and other protein databases typically present functional residues through a protein-centric view, GlyGen’s implementation of ProtVista offers an enhanced, glycosylation-centric perspective. This view integrates glycosylated residues with other critical annotations, including phosphorylation, SNVs, mutagenesis sites, and glycosylation sequons, to highlight their functional interplay. This is unique and necessary for glycobiology research because it allows for the immediate identification of ‘functional overlaps, ’ such as when a glycosylation site resides within a specific binding domain or overlaps, for example, with a phosphorylation residue or SNV, possibly leading to loss of glycosylation and fundamentally altering protein folding or signaling.

This integration allows researchers to explore the protein and glycan annotations together, facilitating a deeper understanding of glycan–protein–function relationships, which otherwise would remain disconnected in datasets and other visualizations. While the current iteration focuses on canonical isoforms, it establishes the architectural foundation to expand visualization to non-canonical isoforms as data become available, ultimately enabling the exploration of the full diversity of the glycoproteome.

The Feature Viewer on the UniProt website uses the ProtVista tool (Fig. 9A) to display the protein sequence features and is accessible through the UniProtKB page for a given protein, and its source code is publicly available as a web component on GitHub: https://github.com/ebi-webcomponents/protvista-uniprot. The ProtVista implemented on GlyGen (Fig. 9B) can be accessed by clicking on the ProtVista button located in the header of the sections such as ‘Glycosylation’, ‘Phosphorylation’, ‘Glycation’, ‘Single Nucleotide Variation’, and ‘Mutagenesis’.

**Figure 9:**
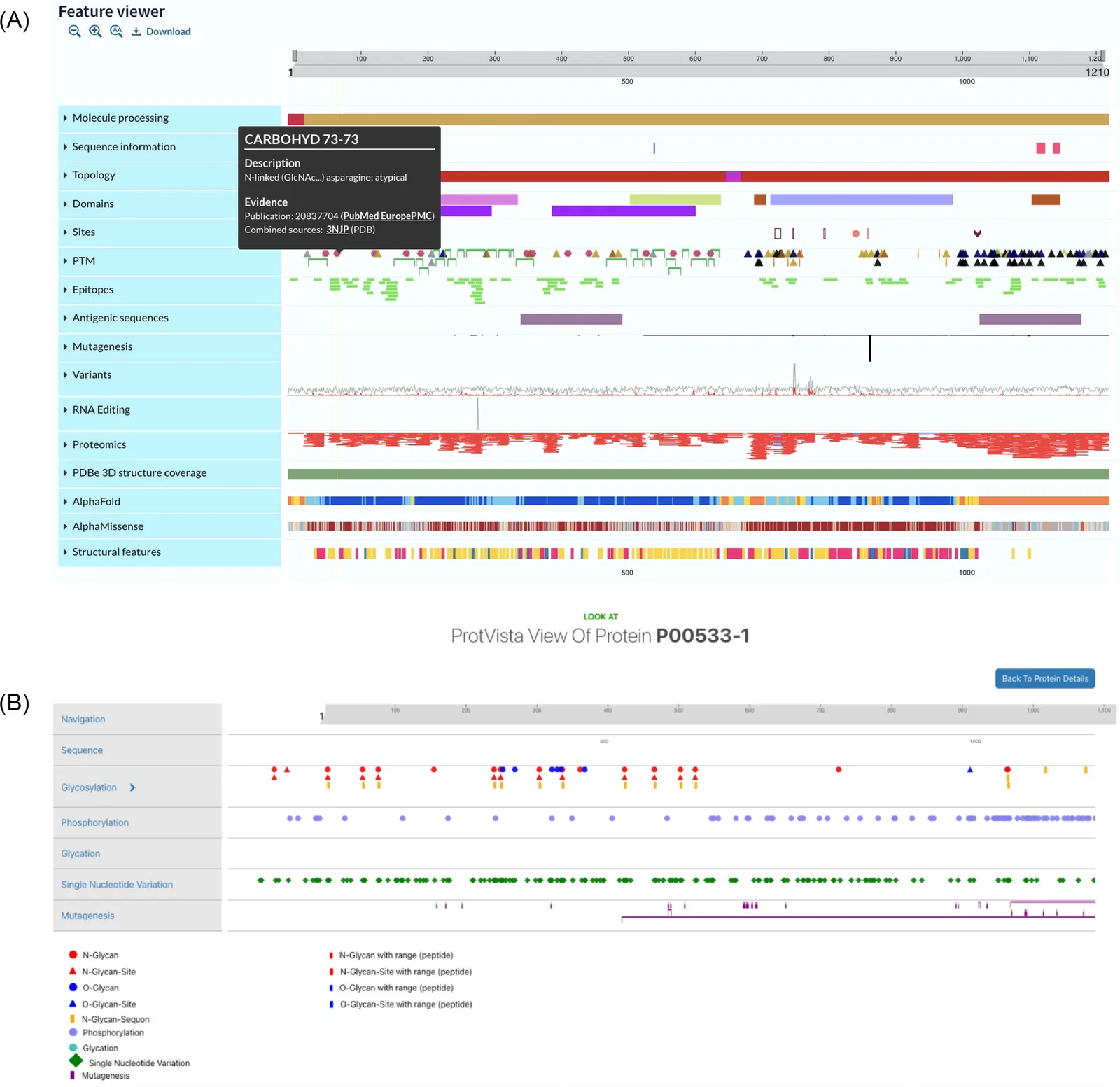
ProtVista Tool view in UniProtKB and GlyGen. (A) UniProtKB ProtVista Feature Viewer for Epidermal growth factor receptor isoform 1 (P00533-1), displaying structural and functional annotations along the sequence. Highlighted features include an atypical *N*-linked glycosylation at Asn73, supported by experimental evidence (PMID:20837704) and PDB data (https://www.uniprot.org/uniprotkb/P00533/feature-viewer). (B) GlyGen-enhanced ProtVista view of Epidermal growth factor receptor isoform 1 (P00533-1) showing glycosylation tracks (*N*-linked glycans, *O*-linked glycans, glycan sequons), phosphorylation, glycation, sequence variants, and mutagenesis sites aligned to the primary sequence.

### Cross-references in GlyGen, UniProtKB, ChEBI, and MatrixDB

Cross-references are essential for enabling data integration, fostering accessibility, and facilitating comprehensive biological research. The interconnection of biological resources improves the discoverability of related data, supports data validation through cross-referencing, and enhances collaborative efforts by uniting interdisciplinary research under a shared data ecosystem. As a result, cross-references accelerate scientific discoveries, promote consistency in analyses, and aid in the development of novel hypotheses in biology and medicine.

GlyGen continuously strives to link proteomics, genomics, and glycomics resources, with cross-references available on protein and glycan detail pages under the Cross-reference section. The protein details page connects to 46 gene–protein-centric resources. These resources provide extensive information on genes, proteins, enzymes, pathways, and more, featuring the ELIXIR core data resources databases like UniProtKB, Reactome, Ensembl, IntAct, ChEBI, Rhea, MatrixDB, ChEMBL, InterPro, Pfam, PRIDE, AlphaFoldDB, EuropePMC, OMA, and QuickGO, whereas the glycan details page connects to 23 glycan-centric databases, including ChEBI, Reactome, Rhea, and MatrixDB.

UniProt contains cross-references to 185 databases to enrich the information provided in UniProtKB protein entries. Cross-referenced databases in UniProt include nucleotide sequence databases such as Ensembl and RefSeq, genetic variation databases including dbSNP, ClinGen, and BioMuta, model organism databases such as HGNC, FlyBase, and MGI, 3D structure databases such as PDB and AlphaFold, PTM databases such as GlyGen and iPTMnet, and various other genomics and proteomics resources. A full list of cross-referenced databases can be found and searched in the ‘Cross-referenced databases’ dataset within the Supporting data section of the UniProt homepage. Explicit and implicit links to these databases are provided in the cross-references section of the UniProtKB entries, and an individual protein entry can contain cross-references to numerous databases and several hundred individual links.

ChEBI provides cross-references to numerous external databases, which are either added manually by a curator or submitter, or added automatically by matching names and/or structures against those present in freely available databases. Cross-references are categorized into protein sequences, reaction and pathways, small molecules, enzymes, gene expression, and molecular interaction resources, and include databases such as UniProt, Reactome, Rhea, PubChem, GlyGen, BRENDA, ChEMBL, and KEGG.

MatrixDB biomolecules contain cross-references to biomolecule data in UniProtKB, ChEBI, the Complex Portal, interaction data in IMEx, IntAct, the Integrated Interactions Database (IID), and expression data in Bgee and proteomic data in MatrisomeDB. Cross-references to GlyGen are currently being added.

## Discussion

The integration of glycobiology data across specialized biological databases represents both a major advance and an ongoing challenge for the glycoscience community. As demonstrated in this manuscript, GlyGen, UniProtKB, ChEBI, MatrixDB, the GlySpace Alliance, and related resources each contribute distinct but complementary layers of information, including protein sequence annotation, glycosylation sites, glycan structures, molecular interactions, chemical identifiers, and cross-references. Together, these resources provide a more connected and interoperable framework for accessing, interpreting, and reusing glycobiology data.

A central strength of this ecosystem is the use of controlled vocabularies, stable identifiers, evidence attribution, and standardized annotation practices. UniProtKB provides curated protein and post-translational modification annotations, GlyGen contextualizes these annotations with glycan-specific information and evidence, ChEBI supports standardized chemical representation of glycans, and MatrixDB captures glycan- and proteoglycan-related molecular interactions. Cross-references among these resources allow users to move between protein-centric, glycan-centric, chemical, and interaction-focused views of the same biological entity, reducing fragmentation and improving data discoverability.

Despite these advances, important challenges remain. Glycosylation is inherently complex because glycans are not directly encoded by the genome, can vary by tissue, cell type, disease state, and experimental context, and often occur as heterogeneous mixtures at the same protein site. This microheterogeneity makes it difficult to represent glycosylation information using simple annotation models. While current approaches are effective for common glycosylation types and experimentally supported sites, additional work is needed to capture ambiguous glycan structures, site occupancy, glycoform diversity, and context-specific glycosylation patterns in a scalable and standardized manner. The rapid growth of high-throughput glycoproteomics data further emphasizes the need for sustainable pipelines that preserve evidence, provenance, and biological context.

The tools and integrations described in this work illustrate how collaborative database development can address practical research needs and support future computational applications. The GlyGen Isoform Mapper helps preserve site-specific annotations reported on non-canonical isoforms, while the implementation of ProtVista in GlyGen enables glycosylation sites to be viewed alongside phosphorylation, glycation, sequence variants, mutagenesis sites, domains, and glycosylation sequons. These harmonized, evidence-linked datasets also create opportunities for artificial intelligence and machine learning approaches to predict glycosylation sites, identify glycan–protein relationships, prioritize experimentally unvalidated annotations, and explore links between glycosylation and disease. Continued expansion of experimentally validated glycosylation data, improved integration of high-throughput datasets, enhanced visualization tools, and sustained cross-resource collaboration will be critical for ensuring that glycobiology data remain findable, accessible, interoperable, and reusable.

## Conclusion

The collaboration among UniProtKB, GlyGen, MatrixDB, ChEBI, and other bioinformatics resources represents a significant step forward in glycobiology research. By providing integrated, high-quality data on protein glycosylation, these platforms enable researchers to explore and understand the complex roles of glycans and glycoproteins in biological processes. Their use of structured annotation methods, controlled vocabularies, stable identifiers, and evidence-based data integration ensures that the information provided is comprehensive, reliable, and interoperable. As glycobiology continues to grow in importance across health, disease, and other areas of biomedical research, this integrated approach will be invaluable for advancing our understanding of glycan structures, glycoprotein function, and their broader implications in cellular biology. Future efforts should focus on enhancing cross-resource data integration, developing more sophisticated analysis and visualization tools, and expanding the coverage of glycan-related information across species, tissues, diseases, and biological contexts.

## Data Availability

Data described in the manuscript are available in GlyGen (https://www.glygen.org), UniProtKB (https://www.uniprot.org), MatrixDB (https://matrixdb.univ-lyon1.fr/), and ChEBI (https://www.ebi.ac.uk/chebi/). The source code for the ProtVista tool is available at https://github.com/ebi-webcomponents/protvista-uniprot.
